# Clinical and Histopathological Assessment of the Field of Cancerization in Human Skin Before and After Treatment With a 1927‐nm Thulium Fractional Laser

**DOI:** 10.1002/lsm.70119

**Published:** 2026-03-01

**Authors:** Livia Maria Oliveira Salviano, André Bubna Hirayama, Marcella Soares Pincelli, Vivian Nunes Arruda, Rodrigo Cesar Davanço, Luiza Reis Pinto, Luis Antonio Ribeiro Torezan

**Affiliations:** ^1^ Department of Dermatology University of São Paulo São Paulo Brazil; ^2^ Department of Pathology University of São Paulo São Paulo Brazil; ^3^ School of Medicine University of Santo Amaro (UNISA) São Paulo Brazil

## Abstract

**Background:**

Field cancerization is a major concern in photodamaged skin due to its risk of progression to squamous cell carcinoma. The 1927‐nm thulium laser has emerged as a promising therapy, but its isolated effects on this condition remain underexplored.

**Objective:**

To evaluate clinical and histopathological changes in field cancerization treated with the 1927‐nm thulium laser.

**Methods:**

It was a prospective, observational, and interventional clinical study. Twenty‐three patients (age average of 61.3 years), phototype I–III, with facial photodamage and multiple actinic keratosis underwent 4 monthly sessions of thulium laser, followed by a 3‐month evaluation. Outcomes included lesion counts and a photoaging scale at each session, along with comprehensive histological analysis of pre‐ and post‐treatment biopsies from 20 patients.

**Results:**

There was a significant reduction in AK count (*p* < 0.001) and photoaging parameters (*p* < 0.001). Histologically, we observed improvement in vertical extension of keratinocyte atypia (*p* = 0.046), increased dermal thickness (*p* = 0.012), and fibroplasia (*p* < 0.001). No severe adverse effects occurred.

**Conclusion:**

The 1927‐nm thulium laser is a safe, well‐tolerated and has promising results as a field‐directed therapy for actinic keratosis, promoting both clinical and histopathological improvement.

## 1 Introduction

Field cancerization refers to the accumulation of genetic mutations and histological alterations in chronically sun‐exposed skin, predisposing it to actinic keratoses (AK) and squamous cell carcinoma (SCC) [1, 2, 3, 4]. First described by Slaughter et al. in 1953 [3], it represents a continuum of subclinical and clinical epidermal and dermal changes typical of photodamaged skin [4]. Photodamage combines clinical signs of photoaging with molecular alterations linked to carcinogenesis [5, 6, 7], including textural, pigmentary, and vascular changes such as wrinkles, lentigines, telangiectasias, and sebaceous gland hypertrophy [5]. AKs—the most common clinical manifestation—consist of intraepithelial atypical keratinocyte proliferation, presenting as normochromic, erythematous, or brownish papules or plaques [7]. The progression of AK to SCC ranges from 0.025% to 16% per year, with contiguity reported in up to 97% of cases [8, 9].

Chronic UV exposure induces molecular alterations, including DNA damage, oxidative stress, and inflammation [8, 9, 10], contributing to carcinogenesis [10, 11]. Field cancerization is clinically relevant due to its malignant potential [12]; areas with multiple AKs show alterations similar to invasive SCC [11, 12, 13]. As a result, management has shifted from treating isolated lesions to addressing the entire field [14].

Field‐directed therapies include topical agents, photodynamic therapy, and laser‐based modalities [13, 14, 15]. Non‐ablative fractional lasers have gained prominence for inducing dermal remodeling while preserving the epidermal barrier, reducing downtime and adverse effects [16, 17, 18, 19, 20].

Although the 1927‐nm thulium laser is classified as non‐ablative, its strong water absorption *(absorption coefficient of water ∼129.2 cm^−1^)* produces superficial energy deposition. At higher fluences and densities, it can induce subepidermal heating or superficial ablation, generating microthermal zones that remove dysplastic keratinocytes while allowing rapid re‐epithelialization. Its penetration depth of 200–300 µm corresponds to epidermal and upper dermal involvement in AKs, supporting observed clinical and histologic improvements [21, 22, 23, 24, 25, 26, 27, 28, 29, 30, 31, 32, 33].

In this study, we evaluated the safety and efficacy of the 1927‐nm thulium laser for treating AKs and photodamaged skin in a prospective clinical and histological trial.

## 2 Objectives

This study aimed to evaluate the clinical and histological changes in the skin of patients with field cancerization before and after treatment with non‐ablative fractional thulium 1927‐nm laser. Furthermore, it sought to *investigated its therapeutic potential and adverse effects as a field cancerization treatment*.

## 3 Patients and Methods

This prospective, observational, and interventional clinical study was approved by the Research Ethics Committee of the Hospital das Clínicas, Faculty of Medicine, University of São Paulo (HCFM/USP), under protocol number CAAE 67372023.9.0000.0068, and conducted in accordance with the Declaration of Helsinki. All participants provided written informed consent prior to enrollment.

The study included patients with Fitzpatrick skin phototypes I–III and clinically significant facial photodamage, recruited between December 2022 and March 2023 from the dermatology outpatient clinics at the Hospital das Clinicas, Faculty of Medicine, University of São Paulo, Brazil.

Eligible participants were at least 18 years old, presented with at least three AK grades I or II [34] on each side of the face, and exhibited clinical signs of photoaging, such as solar lentigines, coarse wrinkles, fine lines, and mottled pigmentation. Patients with a history of photosensitivity disorders, immunosuppression, active infection, a tendency to form keloids, pregnancy, or breastfeeding were excluded, as were those who had received prior laser therapy, photodynamic therapy, or cosmetic procedures within the past 6 months, or used topical agents such as retinoids, 5‐fluorouracil, imiquimod, or diclofenac sodium within the past 3 months. Participants were allowed to withdraw from the study at any point, either by request or at the discretion of the investigator in the event of safety concerns.

Following eligibility screening, participants underwent a baseline skin biopsy 1 week prior to the first laser session. Skin samples were obtained using a 4 mm punch biopsy under local anesthesia with 1% lidocaine. We selected the “normal‐appearing” skin on the field‐cancerized area as the site for skin biopsies, with the aim of avoiding any AK lesion in the preauricular region.

After antisepsis with aqueous chlorhexidine, topical 4% lidocaine was applied for 30 min. Procedures were performed with a non‐ablative fractional 1927‐nm thulium laser device (Lavieen, WONTECH, South Korea). *The parameters were: total energy of 20 mJ/spot*, using a 1.6 cm diameter spot size, center‐to‐center spacing (pitch) of 0.7 mm, corresponding to a density of approximately 204 MTZ/cm². A 20% overlap was applied between pulses. One global pass was applied to the entire face, followed by an additional pass on keratotic areas. Each patient underwent four sessions at 1‐month intervals with identical parameters.

Post‐treatment care was standardized for all participants to ensure consistency, reproducibility, and optimal management of adverse effects. Patients were instructed to cleanse the treated area with a gentle, non‐irritating soap and to apply petrolatum‐based ointment for skin occlusion and barrier repair. In addition, a medium‐potency topical corticosteroid (betamethasone 0.1%) was prescribed twice daily for 7 consecutive days to reduce post‐procedural inflammation. Strict photoprotection was recommended throughout the treatment period and follow‐up, including the daily use of broad‐spectrum sunscreen and avoidance of direct sun exposure, aiming to minimize the risk of post‐inflammatory hyperpigmentation.

Pain intensity was measured using the Visual Analog Scale (VAS), ranging from 0 (no pain) to 10 (worst pain experienced), and adverse events were systematically documented.

Clinical response to treatment was evaluated through standardized digital photography, a modified five‐point photoaging scale [35, 36], and AK lesion counts, performed by two independent dermatologists. Assessments were conducted at the following time points: before the first treatment session (T0), before the second (T1), before the third (T2), before the fourth (T3), and 3 months after the final session (T4).

Clinical improvements were measured according to the modified five‐point photoaging scale described by Zane and Dover [35, 36]. This scale evaluates six parameters of photoaged skin—global photoaging score, mottled pigmentation, facial erythema, fine surface lines, sallowness, and tactile roughness—allowing a standardized assessment of treatment outcomes.

Three months after the final session, a second biopsy was performed 0.5 cm from the first to minimize interference from the healing process.

Samples collected before and after treatment were fixed in 10% formalin, embedded in paraffin, sectioned into 3 µm slides for hematoxylin‐eosin staining and evaluated by two independent dermatopathologists.

Statistical analysis was conducted to evaluate correlations between clinical and histological variables. Non‐parametric Wilcoxon tests were used for continuous data, and the McNemar test was applied for categorical comparisons. A significance level of 5% (p ≤ 0.05) was adopted. All analyses were performed using SPSS software, version 25.0 for Windows (IBM Corp., Armonk, NY, USA).

## 4 Results

### 4.1 Clinical Results

Of the 25 patients enrolled, 2 were lost to follow‐up. Among the remaining 23, 3 declined to undergo biopsy procedures. Consequently, while 23 patients received treatment, only 20 underwent both pre‐treatment and post‐treatment biopsies.

A total of 23 patients completed the four sessions of treatment and underwent clinical evaluation across five time points: T0 (baseline), T1 (before the second session), T2 (before the third session), T3 (before the fourth session), and T4 (3 months after the final session).

Baseline demographic and clinical characteristics of the study population are summarized inTable 1. The mean age was 61.4 ± 11.0 years, and the majority were female (16/23; 70%). Regarding Fitzpatrick phototypes, 48% of patients were classified as phototype II, 35% as phototype III, and 17% as phototype I. No patients with phototype V or VI were included. Baseline severity according to the modified photoaging scale showed that 39% of patients presented with a global score of 3, 30% with a score of 2, and 30% with a score of 4.

**TABLE 1:** Baseline demographic and clinical characteristics.

Variable	Value
Number of patients (completed)	23
Age, mean ± SD (years)	61.4 ± 11.0
Sex	
– Female	16 (70%)
– Male	7 (30%)
Fitzpatrick phototype	
– Type I	4 (17%)
– Type II	11 (48%)
– Type III	8 (35%)
Baseline photoaging score (global)	
– Score 2	7 (30%)
– Score 3	9 (39%)
– Score 4	7 (30%)

**FIGURE 1:**
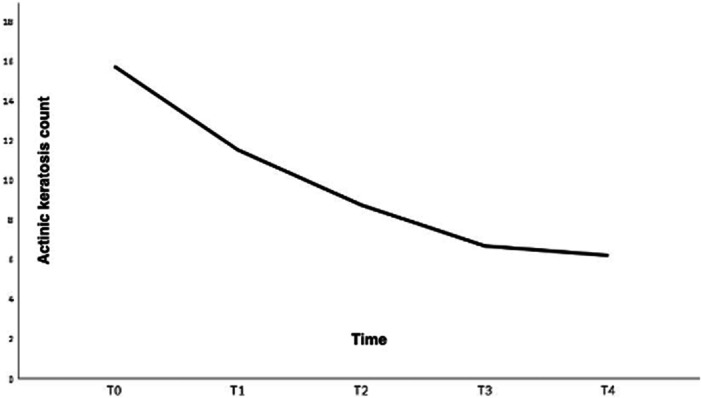
Progressive reduction in actinic keratosis count across clinical evaluation time points (T0–T4).

*The clinical evolution data are shown in* Table 2. The mean number of AK decreased progressively throughout treatment, from 15.8 ± 10.6 at baseline (T0) to 11.5 ± 9.7 at T1 (27% reduction, *p* < 0.001), 8.8 ± 7.5 at T2 (44% reduction, *p* < 0.001), 6.7 ± 6.2 at T3 (58% reduction, *p* < 0.001), and 6.2 ± 7.0 at T4 (61% reduction, *p* < 0.001), compared to the baseline, as shown in Figure 1.

**TABLE 2:** Descriptive statistics (mean and median) for clinical skin variables by evaluation time point (T0–T4) and pairwise comparisons between clinical evaluation time points (T0–T4) for actinic keratosis count and photoaging scale variables. Statistically significant differences (*p* ≤ 0.05, Wilcoxon test) are indicated in bold.

Variable	T0	T1	T2	T3	T4	*p* T0–T1	*p* T0–T2	*p* T0–T3	*p* T0–T4	*p* T1–T2	*p* T2–T3	*p* T3–T4
AK count	15.8 (10.6)	11.5 (9.7)	8.8 (7.5)	6.7 (6.2)	6.2 (7.0)	< 0.001	< 0.001	< 0.001	< 0.001	< 0.001	0.002	0.043
Global score photoaging	3.0 (0.5)	2.9 (0.5)	2.7 (0.6)	2.5 (0.6)	2.5 (0.6)	0.083	0.002	0.001	0.001	0.004	0.063	0.102
Mottled pigmentation	2.3 (0.6)	2.3 (0.5)	1.9 (0.5)	1.7 (0.5)	1.5 (0.6)	0.317	0.001	< 0.001	< 0.001	< 0.001	0.008	0.008
Facial erythema	2.2 (0.7)	2.0 (0.6)	1.6 (0.5)	1.4 (0.5)	1.2 (0.4)	0.053	< 0.001	< 0.001	< 0.001	< 0.001	0.033	0.003
Fine surface lines	2.7 (0.7)	2.6 (0.8)	2.4 (0.7)	2.3 (0.7)	2.1 (0.7)	0.157	0.003	0.001	< 0.001	0.004	0.020	0.020
Sallowness	1.5 (0.7)	1.4 (0.7)	1.0 (0.5)	0.9 (0.5)	0.9 (0.6)	0.034	< 0.001	< 0.001	< 0.001	0.001	0.059	0.157
Tactile roughness	2.4 (0.6)	2.2 (0.6)	1.7 (0.6)	1.4 (0.5)	1.1 (0.6)	0.014	< 0.001	< 0.001	< 0.001	0.001	0.002	0.002

*Note:* Data presented as mean (SD).

**FIGURE 2:**
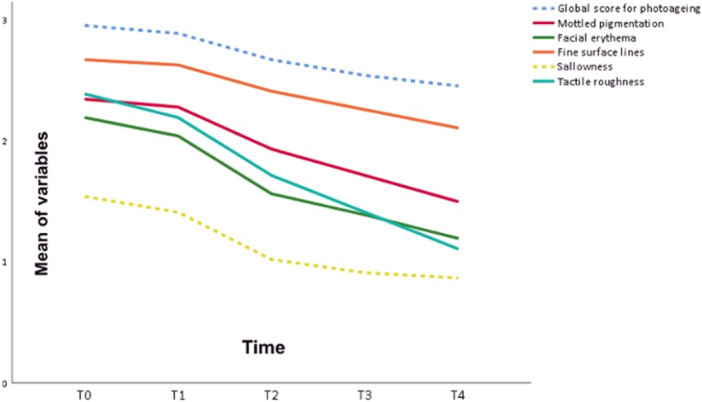
Mean scores of photoaging scale variables across evaluation points (T0–T4). Progressive reductions were observed in most parameters following each thulium laser session, indicating consistent clinical improvement over time.

**FIGURE 3:**
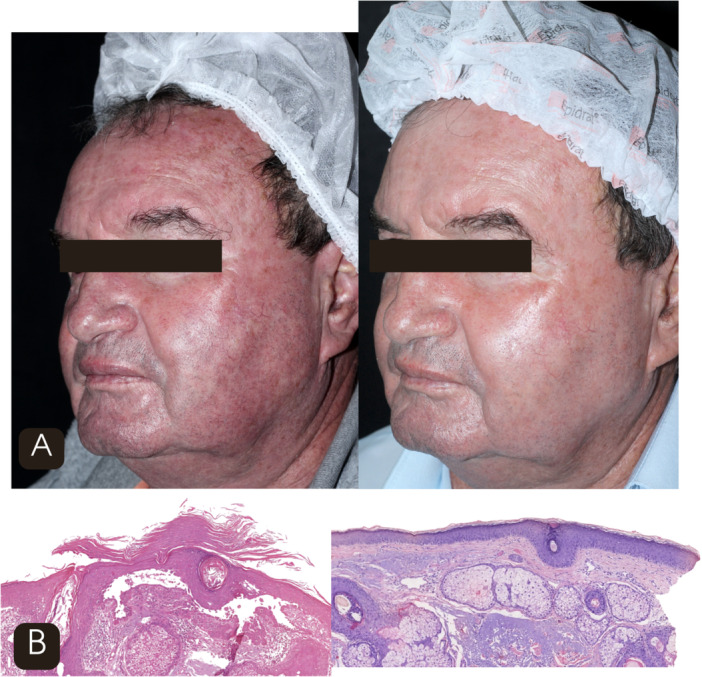
(A) Clinical comparison before (left) and after four sessions (right) of 1927‐nm thulium fractional laser showing reduction in actinic keratoses and improvement in erythema, mottled pigmentation, and skin texture. (B) Histological (H&E 10×) comparison before (left) and after four sessions (right) demonstrating decreased vertical keratinocyte atypia and increased dermal fibroplasia.

Clinical improvement was also documented across multiple parameters of the modified photoaging scale. Mottled pigmentation decreased from 2.3 ± 0.6 at baseline (T0) to 1.5 ± 0.6 at T4 (*p* < 0.001). Erythema improved from 2.2 ± 0.7 to 1.2 ± 0.4 (*p* < 0.001), while fine surface lines decreased from 2.7 ± 0.7 to 2.1 ± 0.7 (*p* < 0.001). Sallowness improved from 1.5 ± 0.7 to 0.9 ± 0.6 (*p* < 0.001), and tactile roughness from 2.4 ± 0.6 to 1.1 ± 0.6 (*p* < 0.001). As illustrated in Figure 2, *the temporal trend of these variables across the five time points, highlighting their progressive reduction throughout treatment*. Figure 3 illustrates the clinical improvement observed after four sessions.

### 4.2 Pain and Adverse Events

**FIGURE 4:**
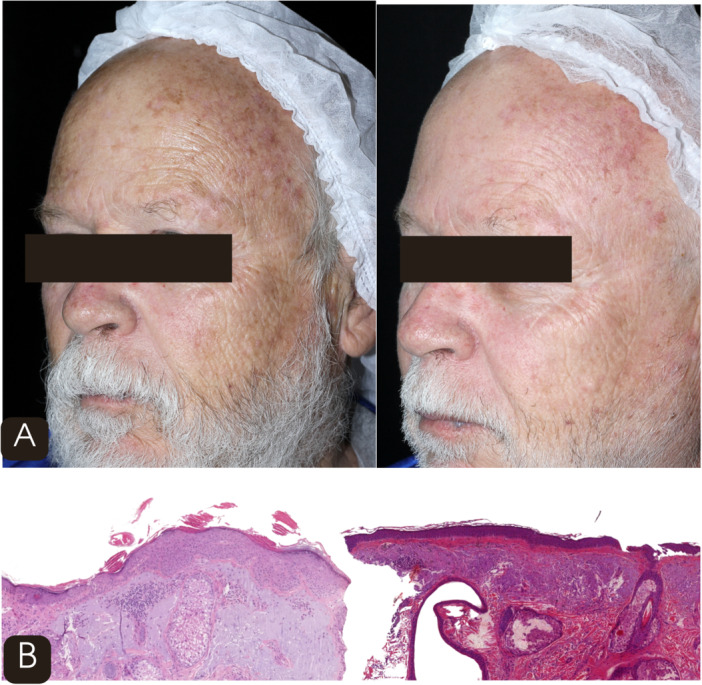
(A) Clinical comparison before (left) and after four sessions (right) of 1927‐nm thulium fractional laser showing reduction in actinic keratoses and improvement in erythema, mottled pigmentation, and skin texture. (B) Histological (H&E 10×) comparison before (left) and after four sessions (right) demonstrating decreased vertical keratinocyte atypia and increased dermal fibroplasia.

**FIGURE 5:**
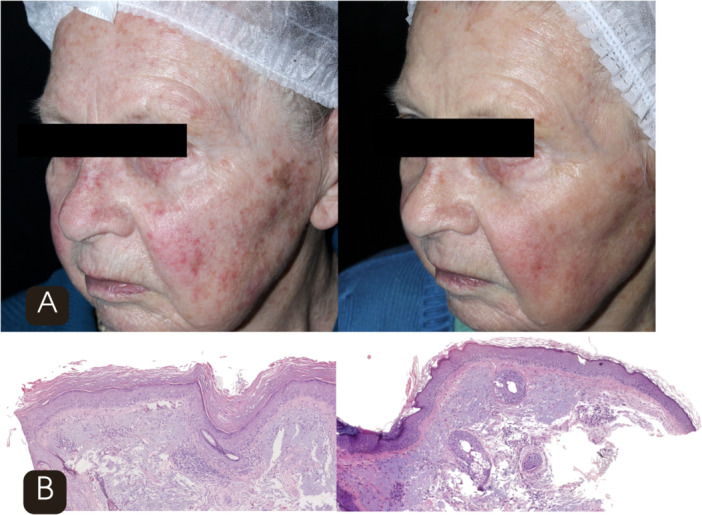
(A) Clinical comparison before (left) and after four sessions (right) of 1927‐nm thulium fractional laser showing reduction in actinic keratoses and improvement in erythema, mottled pigmentation, and skin texture. (B) Histological (H&E 10×) comparison before (left) and after four sessions (right) demonstrating decreased vertical keratinocyte atypia and increased dermal fibroplasia.

Pain was assessed using the Visual Analog Scale (VAS, 0–10). Mean VAS scores were 7.0 ± 1.1 at T0, 7.3 ± 1.0 at T1, 7.3 ± 1.1 at T2, 7.2 ± 1.0 at T3, and 7.3 ± 1.0 at T4, remaining relatively stable across sessions. No severe adverse events were observed. The most common effects were mild and transient erythema, edema, and burning sensation, typically resolving within 48–72 h. *One case of post‐inflammatory hyperpigmentation was observed after the fourth session in a phototype III patient who reported unintentional sun exposure despite photoprotection guidance. The lesion was treated with a triple‐combination topical formulation containing tretinoin (0.1 mg/g), hydroquinone (40 mg/g), and fluocinolone acetonide (0.5 mg/g), applied on alternate days for 2 months. Complete clinical resolution was achieved without recurrence. No infections, persistent erythema, or scarring were observed during the study period* (Figures 4 and 5).

### 4.3 Histopathological Results

Of the 23 patients, 20 underwent paired biopsies before and after treatment. *All biopsies were obtained from clinically lesion‐free, normal‐appearing skin within the field‐cancerized area, deliberately avoiding clinically visible actinic keratoses. For descriptive purposes, we considered baseline samples to show histopathological features consistent with actinic keratosis on H&E sections when keratinocyte atypia and solar elastosis were accompanied by overlying parakeratosis; using these criteria, 7/20 (35%) pre‐treatment biopsies met this definition despite the absence of clinical lesions. The remaining 13/20 (65%) baseline samples demonstrated keratinocyte atypia and solar elastosis but lacked parakeratosis and therefore did not meet full histopathological criteria for actinic keratosis, supporting the presence of subclinical field cancerization*.

Treatment induced a significant reduction in the vertical extension of keratinocyte atypia: lower‐third involvement increased from 60.0% to 75.0%, while extension beyond one‐third decreased from 35.0% to 15.0% (*p* = 0.046). The vertical distribution of atypical keratinocytes was graded according to Röwert‐Huber et al. [37] into three categories: atypia confined to the lower third of the epidermis (AK I), extending to the lower two‐thirds (AK II), or involving more than two‐thirds of the epidermis (AK III). The degree of cytologic atypia was also classified based on nuclear and architectural criteria as mild (slight nuclear enlargement, subtle hyperchromasia, minimal pleomorphism, and preserved epidermal architecture), moderate (nuclear pleomorphism, irregular nuclear contours, increased nuclear‐to‐cytoplasmic ratio, occasional suprabasal mitoses, and partial loss of polarity), or severe (marked pleomorphism, hyperchromasia, prominent nucleoli, frequent atypical suprabasal mitoses, complete architectural disorganization, and dyskeratosis). Based on these definitions, the distribution of atypia grades showed no significant shift, with mild atypia in 70.0% pre‐treatment vs. 65.0% post‐treatment and moderate atypia in 30.0% vs. 25.0% (*p* = 0.405); no cases of severe atypia were observed.

Regarding structural parameters, epidermal thickness decreased significantly from 0.120 ± 0.069 mm to 0.081 ± 0.027 mm (*p* = 0.019), indicating epidermal resurfacing. Dermal thickness, defined as the distance between the dermo‐epidermal junction and the upper limit of the subcutaneous tissue, increased from 1.935 ± 0.712 mm to 2.443 ± 0.551 mm (*p* = 0.012). In this study, fibroplasia thickness was defined as the linear distance from the basal membrane of the epidermis to the end of the neocollagenized area, following the method described by Noronha et al. (2005) [38]. Fibroplasia thickness increased from 0.060 ± 0.075 mm to 0.095 ± 0.055 mm (*p* = 0.005). No significant changes were detected in solar elastosis, which remained intense in 75.0% at baseline vs. 80.0% post‐treatment (*p* = 0.655).

## 5 Discussion

This study demonstrated that treatment of photodamaged skin and field cancerization with the 1927‐nm non‐ablative thulium laser is both safe and effective. *Rather than presenting abrupt changes, the treatment showed a gradual, cumulative reduction in AK lesions across sessions, suggesting that most of the therapeutic gain is achieved within the first three sessions, with incremental benefit from completing the full course.* These results are in line with Weiss et al. [1], who reported 86.6% AK clearance 6 months after 1927‐nm treatment.

Beyond visible lesions, the therapeutic effect extended to the broader field of photodamage. *Improvements in multiple domains of the photoaging scale support the notion that this modality influences not only actinic keratoses but also textural and pigmentary signs of aging*. Notably, parameters such as global photoaging score and sallowness appeared to stabilize earlier, indicating potential saturation of effect in these aspects and suggesting that some benefits may plateau before the final session. These findings highlight the potential dual value of the thulium laser in targeting both carcinogenic potential and cosmetic aspects of photodamage.

Although most studies have focused on clinical outcomes, our study adds a novel contribution by incorporating histopathological analysis, offering a more comprehensive view of tissue remodeling and cancerization reversal. *The use of paired biopsies in clinically lesion‐free skin enabled objective assessment of subclinical field repair.* Few studies in the literature have systematically addressed histological changes in field cancerization treated with the 1927‐nm thulium laser, particularly with objective dermal and epidermal metrics in subclinical areas. This strengthens the value of our findings in bridging clinical response with histological evidence of field repair [25, 26].

Importantly, the identification of histopathological features consistent with actinic keratosis in clinically normal‐appearing skin underscores the dissociation between clinical inspection and the true burden of field cancerization. This finding reinforces the rationale for field‐directed approaches, as subclinical epidermal alterations may persist even in areas without visible lesions, and supports histology as a complementary endpoint beyond clinical lesion counting [5, 13].

Histopathological assessment corroborated the clinical findings. *The observed reduction in the vertical extension of keratinocyte atypia, despite a relatively stable grading of severity, points to a biological shift toward re‐epithelialization and reduced dysplasia depth*. These findings parallel those of Szeimies et al. [13], who described attenuation of atypia after field‐directed photodynamic therapy, emphasizing the role of histological endpoints in evaluating efficacy.

Changes in tissue architecture further support the regenerative role of the 1927‐nm laser. Epidermal thinning likely reflects selective ablation of atypical keratinocytes, while dermal thickening and fibroplasia indicate new collagen formation. This dermal fibroplasia may represent the histologic counterpart of the subepidermal low‐echogenic band (SLEB) observed in high‐frequency ultrasound studies [35, 38]. These results align with previous reports on collagen remodeling and dermal neogenesis following thulium laser therapy [27, 28, 29]. *In contrast, persistent solar elastosis in most patients suggests that advanced photodamage may be less responsive in the short term and could benefit from adjuvant or ablative strategies. Because elastin degeneration is generally considered poorly reversible, persistence of solar elastosis on H&E‐stained sections was expected; no elastic fiber‐specific staining was performed, which may limit detection of subtle qualitative changes* [35].

When compared with other field‐directed therapies, the role of the 1927‐nm thulium laser appears promising but requires careful contextualization. Current international guidelines recommend field treatments such as 5‐fluorouracil (5‐FU), imiquimod, and photodynamic therapy (PDT) as first‐line options, supported by the strongest levels of evidence [5]. Ablative lasers such as CO₂ and Er:YAG are also mentioned in some guidelines, although with lower evidence levels and mainly as alternative approaches. Importantly, ablative laser procedures are not recommended for immunosuppressed or organ transplant recipients due to their higher risk of complications and limited safety data in these populations [6, 7, 8]. Non‐ablative devices, such as the 1550‐nm erbium glass laser, have demonstrated more modest outcomes [9]. Among topical options, 5‐FU and imiquimod achieve clearance rates often exceeding 70%–80%, but require prolonged application and are associated with intense local inflammation and discomfort. PDT is also effective but limited by procedure‐related pain and recurrence over time. In comparison, the 1927‐nm thulium laser in our study achieved a 61% reduction with relatively few sessions and additional histological evidence of tissue remodeling. While the clearance rate is lower than that of some established therapies, the shorter duration of treatment, dual cosmetic and oncologic benefits, and favorable tolerability profile suggest that it may represent a valuable alternative in selected patients [27, 28, 29]. *A recent systematic review further supports this perspective, highlighting consistent efficacy of the 1927‐nm thulium laser across dermatological indications, including photodamage, with favorable outcomes in texture, pigmentation, and safety. The review reinforces the role of the water chromophore and superficial thermal effects as central to its dual therapeutic action* [39].

This dual benefit—targeting both clinically evident and subclinical disease—aligns with the therapeutic rationale for field‐directed treatment. As emphasized by international guidelines for the management of AK [5], field cancerization is a dynamic and histologically active process that requires therapies capable of addressing widespread epidermal dysplasia. The histological improvements observed in our study—particularly the reduction in atypia and increase in fibroplasia—reinforce the potential of the 1927‐nm thulium laser to simultaneously achieve oncologic and cosmetic goals [30, 31]. In addition to direct keratinocyte removal, this wavelength may activate paracrine or immunological mechanisms, described as the thermal bystander effect [1], that could contribute to sustained clearance.

The observed reduction in epidermal thickness likely reflects the selective ablation of atypical keratinocytes, followed by regeneration from relatively preserved stem cell niches such as follicular units. This process has been proposed as a key mechanism in fractional resurfacing and rejuvenation [1]. The increase in fibroplasia, consistent with neocollagenesis, further highlights the cosmetic benefits of this modality. These changes align with prior histological studies of photodamage interventions defining fibroplasia as the extent of neocollagenized dermis [35].

Despite these promising outcomes, some limitations must be acknowledged. The sample size was relatively small, and although statistical significance was reached, the results should be interpreted with caution. The absence of a control group and the relatively short follow‐up (3 months after the last session) limits assessment of long‐term efficacy and recurrence. Randomized or split‐face designs could strengthen future research, though ethical and practical challenges exist when withholding treatment from visibly photodamaged skin. Furthermore, all patients were Fitzpatrick phototypes I–IV, which may limit generalizability to darker skin populations.

Nonetheless, our findings suggest the use of the 1927‐nm thulium laser as a safe, effective, and well‐tolerated modality for treating field cancerization and associated photoaging. The combination of clinical and histopathological improvements reinforces its value as a field‐directed therapy capable of addressing both visible lesions and subclinical dysplasia. This dual action is particularly relevant in dermatologic oncology, where balancing cancer prevention and cosmetic outcomes is crucial. Future studies with larger and more diverse cohorts, longer follow‐up, and incorporation of immunohistochemical or molecular markers are warranted to further elucidate mechanisms of response and predictors of long‐term efficacy. *Future studies may also benefit from incorporating noninvasive imaging modalities—such as reflectance confocal microscopy or optical coherence tomography—to complement histology and enable longitudinal assessment of subclinical field cancerization in vivo* [32].

## 6 Conclusion

The 1927‐nm non‐ablative thulium laser demonstrated significant clinical and histopathological efficacy in the treatment of field cancerization, promoting reductions in AK and photoaging parameters, alongside histological improvements in keratinocyte atypia and dermal remodeling. The treatment proved to be safe, well‐tolerated in this group of patients. While further studies with broader populations and longer‐term follow‐up are warranted, our results suggest this modality as a promising alternative for managing AK and preventing progression to invasive disease.

## Funding

The authors received no specific funding for this work.

## Ethics Statement

This study was approved by the Institutional Review Board of the University of São Paulo, under protocol CAAE 67372023.9.0000.0068. All procedures adhered to the Declaration of Helsinki.

## Consent

Written informed consent was obtained from all participants for participation, clinical photography, and tissue sampling.

## Conflicts of Interest

The authors declare no conflicts of interest.

## Data Availability

The data that support the findings of this study are available from the corresponding author upon reasonable request.
